# *KCTD12* is negatively regulated by Kit in gastrointestinal stromal tumors

**DOI:** 10.18632/oncotarget.25469

**Published:** 2018-06-05

**Authors:** Yoshiyuki Suehara, Keisuke Akaike, Kenta Mukaihara, Aiko Kurisaki-Arakawa, Daisuke Kubota, Taketo Okubo, Hiroyuki Mitomi, Keiko Mitani, Michiko Takahashi, Midori Toda-Ishii, Youngji Kim, Yu Tanabe, Tatsuya Takagi, Takuo Hayashi, Kaoru Mogushi, Kazuo Kaneko, Takashi Yao, Tsuyoshi Saito

**Affiliations:** ^1^ Department of Orthopedic Surgery, Juntendo University School of Medicine, Tokyo, Japan; ^2^ Department of Human Pathology, Juntendo University School of Medicine, Tokyo, Japan; ^3^ Center for Genomic and Regenerative Medicine, Juntendo University School of Medicine, Tokyo, Japan

**Keywords:** gastrointestinal stromal tumor, KIT, pfetin, mutation, tumor suppressor

## Abstract

Our group has previously demonstrated that pfetin, encoded by the *KCTD12* gene, is a strong prognostic biomarker for gastrointestinal stromal tumors (GISTs). However, the underlying mechanisms that control pfetin expression remain unknown. To elucidate the regulatory mechanisms of *KCTD12* in GIST, in addition to a possible association between *KCTD12* alterations and protein expression, we examined 76 patients with GISTs for *KCTD12* mutations by PCR-direct sequence, and compared these results with clinicopathologic data. The function of pfetin in GIST progression was also revealed using GIST T1 cells. In this series, pfetin expression was not observed in 15 cases, and loss of pfetin expression was associated with higher mitotic rate (>5/50HPFs: *p* = 0.029). There was also a trend between presence of necrosis and loss of pfetin expression but this was not statistically significant (*p* = 0.09). *KCTD12* mutations were frequently observed in 22 out of 76 GISTs (28.9%); however, they did not affect protein expression and were not associated with patients’ prognosis. *KCTD12 in vitro* knockdown resulted in the accelerated growth of GIST T1 cells, confirming that pfetin functions as a tumor suppressor. *KIT* knockdown significantly inhibited cellular growth and upregulated the expression of pfetin at both the mRNA and protein level. These findings suggest that GISTs with loss of pfetin expression has proliferative advantage and that higher pfetin expression in GISTs may be indicative of lower expression levels of *KIT*. This relationship confirms that pfetin is a useful prognostic marker in GISTs.

## INTRODUCTION

Although gastrointestinal stromal tumors (GISTs) comprise <1% of all gastrointestinal tumors occurring in patients, they are the most common primary mesenchymal tumor of the digestive tract, with a prevalence of 15 to 20 per 1,000,000 [1, 2]. GISTs arise predominantly in the stomach (60% of cases), small intestine (25%), rectum (5%), esophagus (2%), and various other locations [3]. GISTs are characterized by activating mutations in genes encoding the KIT Proto-Oncogene Receptor Tyrosine Kinase (KIT) or the platelet-derived growth factor receptor alpha (*PDGFRA*) [3]. Pfetin (potassium channel tetramerization domain containing 12), encoded by the *KCTD12* gene, was identified as an auxiliary subunit of GABA_B_ receptors that directly influences the biophysical and pharmacological properties of the receptor responses [4, 5]. Using a proteomic approach, our group has previously reported that pfetin is a strong prognostic biomarker for GIST [6], and this conclusion has been confirmed by numerous several follow-up studies [7–9]. Although the specific function of pfetin in GIST tumorigenesis and progression remains unknown, it is likely that pfetin has an important tumor-suppressive role in GISTs. Numerous genetic analyses of tumor suppressor genes have been performed, and correlations between the mutational status, risk of cancer, prognostic outcome, and chemo-sensitivity have been thoroughly detailed [10–14]. Furthermore, genetic alterations in specific regions of the tumor suppressor genes/oncogenes are known to change molecular structures and affect their function of the encoded protein [15, 16]. Therefore, the ability to correlate genetic mutations with clinical information is an important strategy to characterize genes whose functions remain unknown. Recently, an integrated genomic analysis of ovarian carcinoma revealed a genetic mutation in *KCTD12* in one case of high-grade serous ovarian adenocarcinoma (HGS-OvCa) [17]. The prognostic value of *KCTD12* mutations in serous OvCa remains unknown, as the *KCTD12* mutation was detected only in a single case of a large HGS-OvCa study cohort. It is therefore pertinent to examine the genetic alterations of *KCTD12* in GISTs and to compare these with protein expression levels and patient prognosis, because high expression of pfetin correlates with a favorable prognostic outcome. In this study, we first investigated the genetic alterations of *KCTD12* in GISTs to ascertain their clinical impact upon patient survival. In addition, we also performed an analysis of pfetin function to elucidate its hypothesized tumor suppressive role, with a particular emphasis on its relationship to *KIT* expression.

## RESULTS

### *KCTD12* mutations frequently occur in GIST; however, their presence does not affect patient prognosis

In the cohort of GIST patient cases examined in this study, pfetin expression significantly affected the disease-free survival and overall survival of the patients as we previously demonstrated (Supplementary Figure 1A–1D). In this series, pfetin expression was judged as negative in 15 cases (Supplementary Table 1), and loss of pfetin expression was associated with higher mitotic rate (>5/50HPFs: *p =* 0.029 Supplementary Table 2). There was also a trend between presence of necrosis and loss of pfetin expression, but this was not statistically significant (*p =* 0.09). It is known that KIT downstream signal activity is different depending on *KIT* genotype, however, no correlation was observed between *KIT* genotype and pfetin expression level. Furthermore, pfetin expression status did not correlate with immunohistochemically determined c-kit expression level (Supplementary Table 2).

**Figure 1:**
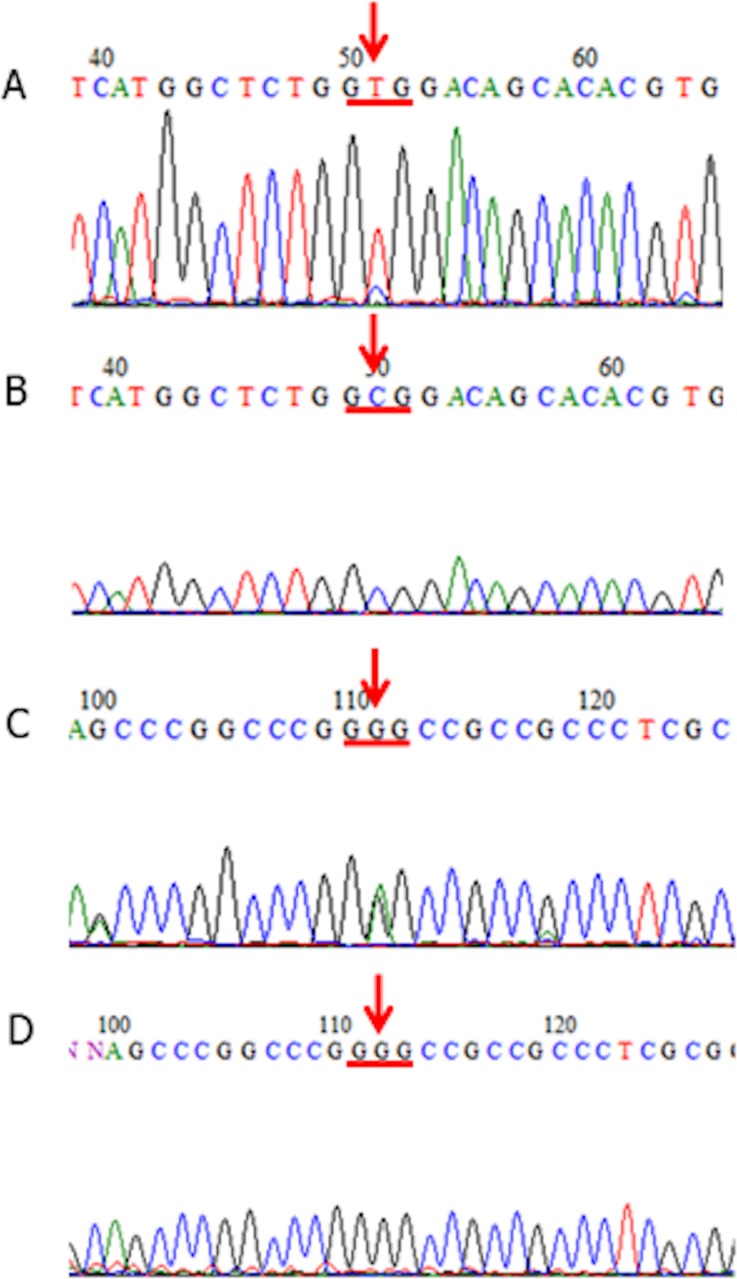
*KCTD12* mutations in GISTs (**A**, **B**) A patient case of GIST (Case #75) that harbored a mutation at codon 4 of the *KCTD12* gene (GCG (Ala) to GTG (Val)) (A: tumor-derived DNA, B: corresponding normal tissue-derived DNA). The tissue samples from this patient case showed immunohistochemically detected pfetin expression (not shown). (**C, D**) A patient case of GIST (Case #32) that harbored a mutation at codon 138 of the *KCTD12* gene (GGG (Gly) to GAG (Glu)) (C: tumor-derived DNA, D: corresponding normal tissue-derived DNA). The tissue samples from this patient case showed immunohistochemically detected pfetin expression (not shown).

We detected many instances of *KCTD12* mutations as somatic mutations (Table 1). In total, 35 *KCTD12* mutations were found in samples of 22 out of 76 patients (Figure 1A–1D), among which only three cases showed decreased pfetin expression. All mutations were missense mutations, and no frameshift or nonsense mutations were identified. The presence of *KCTD12* mutations did not correlate with the immunohistochemically determined pfetin expression level or with the tumor location (expression level; *p =* 0.923 Supplementary Table 2, tumor location; *p =* 0.870 Supplementary Table 3). Furthermore, the presence of *KCTD12* mutations did not affect patients’ overall or recurrence-free survival (data not shown).

**Table 1:** *KCTD12* mutations in GISTs

Patient^#^	Pfetin IHC	*KCTD12* mutation
6	(+)	Codon134 (CAG to TAG)
13	(−)	Codon160 (GGC to AGC)
17	(+)	Codon136 (GGC to GAC)
		Codon138 (GGG to GAG)
25	(−)	Codon136 (GGC to GAC)
26	(−)	Codon141 (CCC to CTC)
		Codon140 (CCG to CTG)
27	(+)	Codon270 (TAT to TGT)
		Codon139 (CCG to TCG)
30	(+)	Codon150 (GGC to GAC)
		Codon245 (GCC to CCC)
31	(+)	Codon138 (GGG to GAG)
32	(+)	Codon132 (CCC to CTC)
		Codon248 (GTG to ATG)
33	(+)	Codon124 (GAG to AAG)
		Codon228 (GCC to ACC)
		Codon272 (CTC to TTC)
34	(+)	Codon139 (CCG to CTG)
35	(+)	Codon131 (GCG to ACG)
		Codon240 (GGA to GAA)
		Codon28 (GAG to GGG)
39	(+)	Codon40 (GTG to ATG)
		Codon134 (CAG to TAG)
		Codon143 (CGG to TGG)
45	(+)	Codon138 (GGG to AGG)
		Codon195 (CTC to TTC)
		Codon206 (CGC to TGC)
46	(+)	Codon191 (GCG to GTG)
54	(+)	Codon185 (AGT to AAT)
60	(+)	Codon126 (GTG to ATG)
63	(+)	Codon132 (CCC to CTC)
65	(+)	Codon134 (CAG to CAT)
73	(+)	Codon14 (GGC to AGC)
74	(+)	Codon4 (GCG to GTG)
77	(+)	Codon123 (CGC to CTC)

### Knockdown of *KCTD12* accelerated cell growth in the GIST T1 cell line

**Figure 2:**
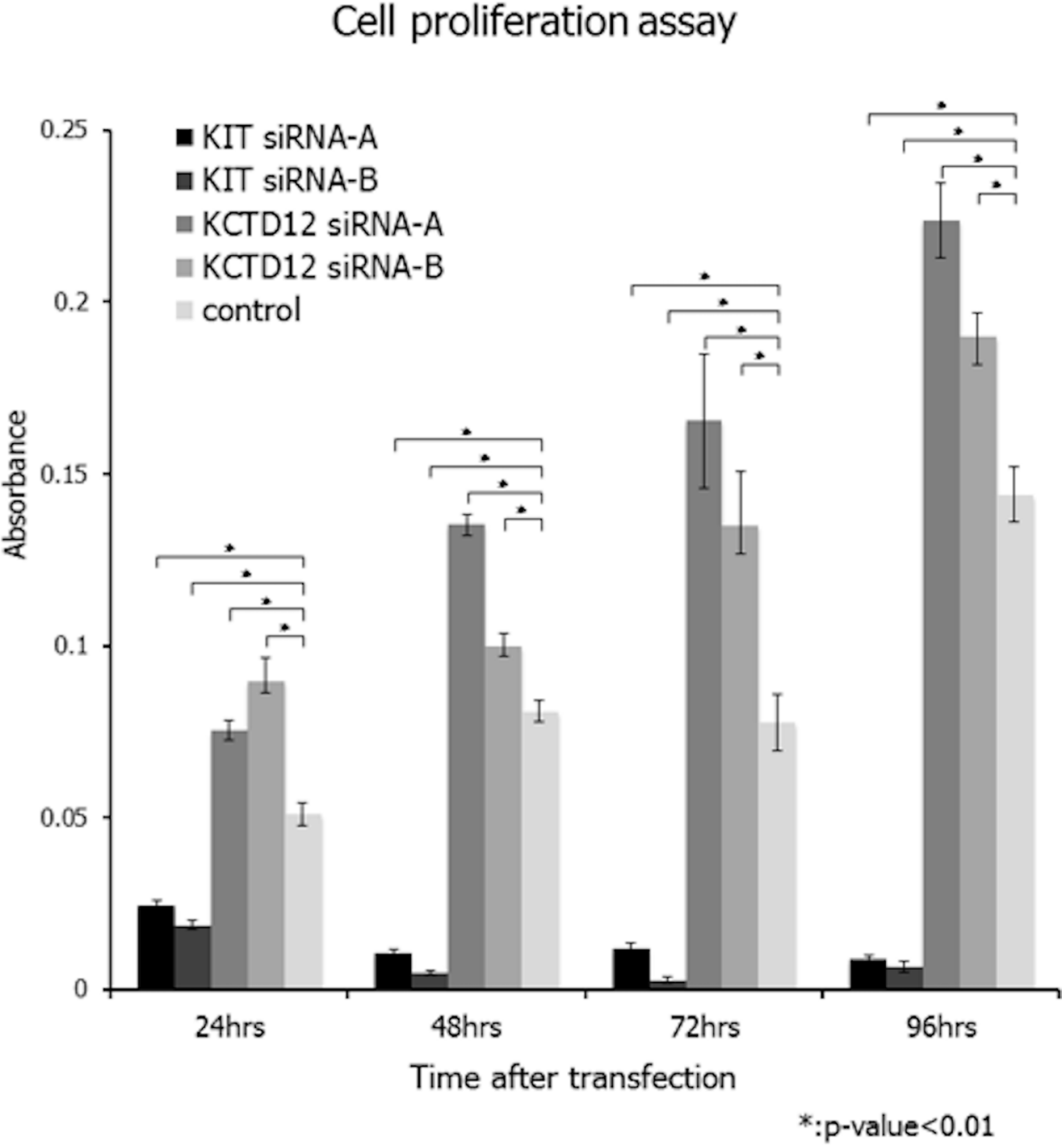
Effects of *KCTD12* and *KIT* knockdown in GIST T1 cells Knockdown of *KCTD12* in the GIST T1 cell line resulted in significantly increased cell proliferation rates at 24–96 h after transfection. Furthermore, *KIT* knockdown was also performed in T1 cells. As expected, the knockdown of *KIT* drastically decreased T1 cell proliferation at 24–96 h after transfection.

First, we confirmed that the GIST T1 cell line did not harbor any genetic alterations of *KCTD12*. We successfully knocked down *KCTD12* expression in the GIST T1 cell line by using two different siRNAs. We found that following *KCTD12* knockdown, cell proliferation rates were significantly increased in the period from 24 to 96 h after transfection (Figure 2). This finding suggests a possibility that pfetin has a tumor suppressor function and controls proliferation of the GIST T1 cells. However, knockdown of *KCTD12* by two siRNAs did not affect GIST T1 cell invasiveness (data not shown). Furthermore, *KIT* knockdown was also performed in the GIST T1 cells. As expected, this manipulation drastically decreased T1 cell proliferation (Figure 2).

### KIT knockdown increased KCTD12 expression at both the mRNA and protein level in the GIST T1 cell line

**Figure 3:**
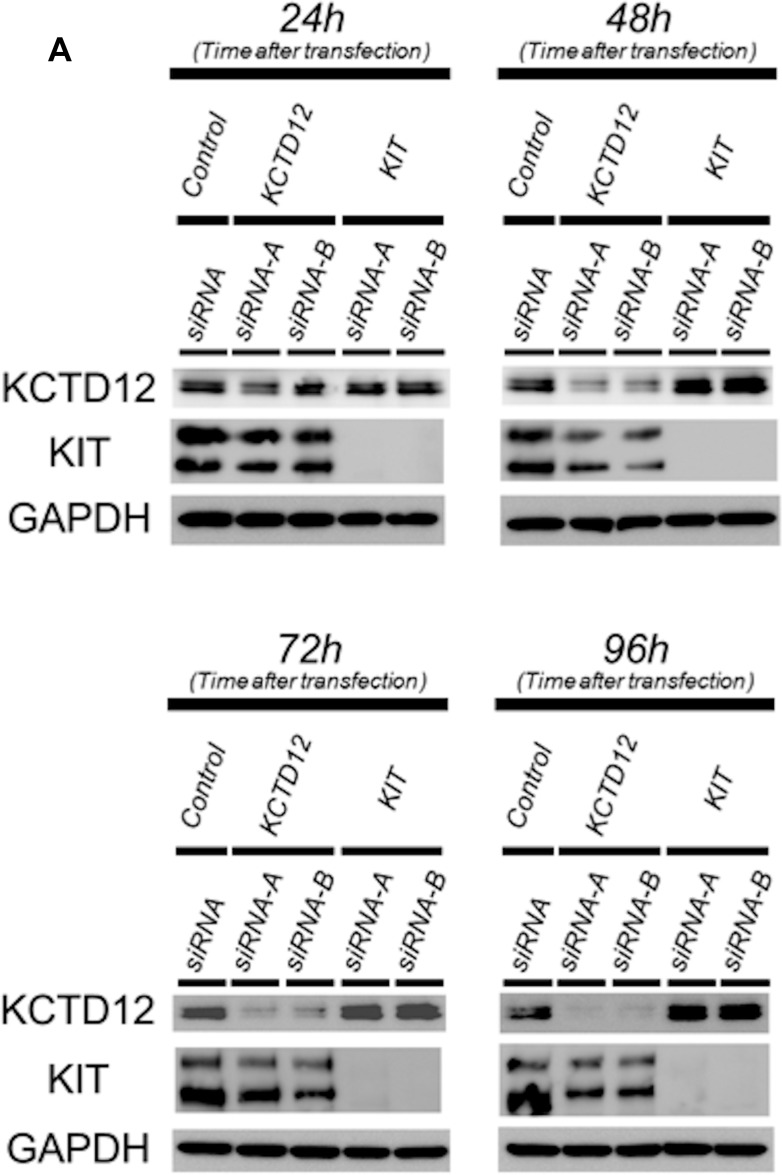

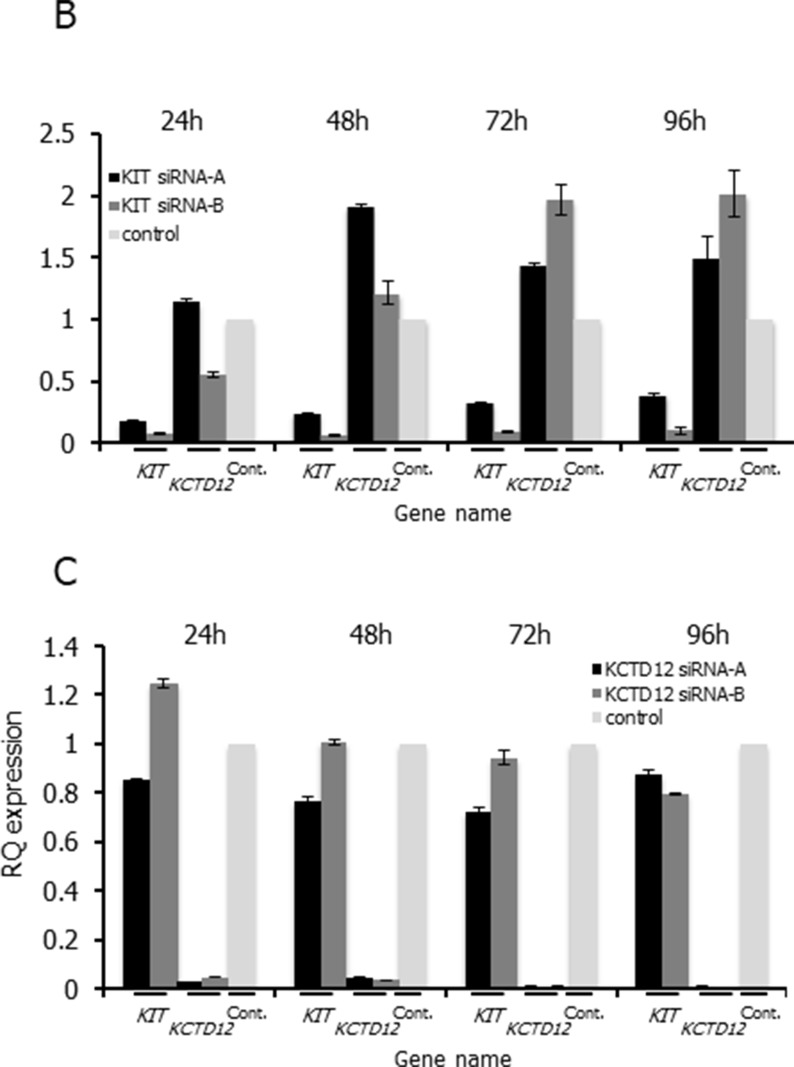
The relationship between the KIT and KCTD12 protein expression levels in GIST T1 cells (**A**) Western blotting was performed at 24–96 h after transfection with corresponding siRNAs. Protein expression levels of KCTD12 increased at 24–96 h after *KIT* knockdown. In contrast, the knockdown of *KCTD12* reduced KIT protein expression in the same period. (**B**) Expression levels of *KCTD12* mRNA increased approximately 1.5–2-fold at 72–96 h after the transfection with *KIT* siRNA. (**C**) Expression levels of *KIT* mRNA showed slight but gradual reduction after the transfection with *KCTD12* siRNA.

To determine the relationship between KIT and *KCTD12* in GIST, we first performed knockdown of *KIT* in the GIST T1 cell line. Both protein (Figure 3A, Supplementary Figure 2) and mRNA (Figure 3B) expression levels of *KCTD12* were significantly increased by the knockdown of *KIT*. In turn, the knockdown of *KCTD12* seemed to reduce the protein expression level of KIT only slightly (Figure 3A, Supplementary Figure 2). The decrease in the *KIT* mRNA level was also minimal, although it was statistically significant (Figure 3C). Global gene expression changes (72 h after siRNA transfection) following knockdown of *KIT* or *KCTD12* were assessed by the microarray analysis (Affymetrix GeneChip Human Genome U133 Pus 2.0) and this inverse relationship was confirmed regarding *KIT* and *KCTD12* expression levels. These results are included in the supplementary data section. These findings suggest a model, in which KIT negatively regulates *KCTD12*.

### Microarray analysis

Global gene expression changes after *KIT* knockdown were larger than that in the case of *KCTD12* knockdown (Supplementary Table 4). As expected, many genes involved in the cell cycle were downregulated by *KIT* knockdown, reflecting a drastic decrease in cell proliferation. In contrast, genes involved in the regulation of cell adhesion and extracellular matrix were upregulated by *KIT* knockdown. A finding of increase of the *KCTD12* mRNA expression by real-time PCR after *KIT* knockdown was confirmed by this microarray analysis, although it was slight (ratios: 1.16 and 1.05). Knockdown of *KCTD12* upregulated the genes associated with extracellular matrix, chemotaxis and lipid metabolism and downregulated genes associated with translational regulations such as RNA modification and processing. A slight decrease in *KIT* mRNA expression after *KCTD12* knockdown observed in real-time PCR assay was also confirmed in this microarray analysis (ratio: 0.93).

## DISCUSSION

Pfetin is an auxiliary GABA_B_ receptor subunit that distinctly influences the biophysical and pharmacological properties of the receptor response [4, 5]. Left–right differences in habenular neuropil formation in the brain are closely correlated with asymmetric expression of pfetin, and a *KCTD12* mutation has been found to cause excess neuropil elaboration [18]. However, the role of pfetin in tumorigenesis and tumor progression has not been previously described. We have previously demonstrated the prognostic value of pfetin expression in GISTs, and validation studies from numerous clinical facilities have confirmed that pfetin expression is a reliable prognostic biomarker [7–9]. In the present study, pfetin expression also significantly correlated with the duration of disease-free survival of GIST patients.

Although we have demonstrated a decreased pfetin expression in a subset of GISTs with poor clinical outcomes, the mechanisms that regulate pfetin expression remain unclear. In a genome-wide mutation screening, a mutation of *KCTD12* has been reported in a case of high-grade serous ovarian carcinoma [17]. In the process of acquiring highly malignant properties, such as metastasis, invasion, and peritoneal dissemination, GISTs are thought to exhibit secondary genetic alterations [19]. We hypothesized that *KCTD12* mutations might be associated with a loss of the tumor suppressor function of pfetin, leading to the acquisition of aggressive phenotypes by the tumor, similarly to the phenomenon that occurs with other tumor suppressor genes, e.g., *CDH1* and *TP53* [20, 21]. *KCTD12* mutations were frequently observed in GIST patients. However, they were not associated with immunohistochemically determined changes in pfetin expression. Furthermore, they did not affect the prognosis of GIST patients. In this study, all changes in the *KCTD12* sequence were missense mutations, and no frameshift or nonsense mutations were identified. This finding might explain, in part, intact pfetin expression in GIST samples. In addition, tumor heterogeneity might also partially account for this observation. We defined pfetin expression as immunohistochemically positive when more than 20% of tumor cells were stained for pfetin [6]. Therefore, it is possible that these mutations were limited to pfetin-immunohistochemically-negative tumor cells that indeed failed to express pfetin. Furthermore, pfetin switching by epigenetic regulation of *KCTD12* may contribute to the level of overall pfetin expression.

It has been shown previously that approximately two-thirds of GISTs with *KIT/PDGFRA* mutations show either monosomy 14 or partial loss of chromosome 14q [22–24]. At the same time, tumor suppressor genes that are important in early GIST development are thought to be located within this region [23, 25]. A loss of the long arm of chromosome 22 is observed in approximately half of all GIST cases and is associated with the progression to borderline/malignant GIST [23, 26–28]. In contrast, a loss of the long arm of chromosome 13, where *KCTD12* is located, is seldom reported [25, 26, 28–30]. To elucidate associations between *KCTD12* mutations and KIT expression, we performed clinicopathological analyses using *KCTD12*/pfetin (mutation and protein expression), *KIT* (protein expression and genotype) and the other pathological factors. With respect to pfetin expression, pfetin expression showed no statistically significant associations with KIT expression level (+ vs 2+/3+) (*p =* 1.000), *KIT* genotype (*p =* 0.706) and risk classification (*p =* 0.651) (Supplementary Table 2). Only higher mitotic index (>5/50HPFs) was associated with decreased pfetin expression (*p =* 0.029). Regarding *KCTD12* mutation, we found no statistically significant associations (KIT expression level (*p =* 0.608), *KIT* genotype (*p =* 0.918), Mitosis index (*p =* 0.789), size (*P =* 0.685), necrosis (*p =* 0.666) and risk classification (*p =* 0.084)) (Supplementary Table 3). In addition, our findings suggest that decreased pfetin expression in GIST is not associated with the putative loss of function caused by genetic alterations, because there was no correlation between KCTD12 mutation and decreased pfetin expression.

To elucidate the tumor suppressor function of pfetin and possible regulatory mechanisms of *KCTD12* in GIST further, we employed RNA interference in cultures of GIST T1 cells. As expected, the cell proliferation rates increased following *KCTD12* knockdown in GIST T1 cells, pointing to a possible mechanism, by which pfetin expression confers a favorable patient prognosis in GIST. This finding is consistent with clinicopathological correlation between loss of pfetin expression and higher proliferative activity in our series of GISTs. Furthermore, knockdown of *KIT* drastically decreased cell proliferation, confirming the central role for KIT in the tumorigenesis of GISTs. GISTs usually present as macroscopically well-circumscribed tumors, although vascular, but not lymphatic, invasions are observed in a subset of cases [31]. The vascular invasion observed in these tumors is associated with liver metastasis and has an adverse prognostic impact for GIST patients [31]. In the present study, we did not observe a clear relationship between pfetin expression and GIST invasiveness. However, it would be too early to conclude that pfetin is not involved in the tumor invasion process. Type III collagen is the main component of collagen in the vascular wall, whereas the invasion assay employed in this study measures the activity of matrix metalloproteinase-2 and matrix metalloproteinase -9, which mainly target type IV collagen predominantly expressed in the basal membrane.

The *KIT* mutations that lead to overexpression of the protein are “driver mutations” in GISTs. Nonetheless, proteomic analysis revealed that pfetin expression is a strong prognostic factor for GISTs. However, the relationship between expression levels of these two proteins remains unknown. Notably, knockdown of *KIT,* which decreased the cell proliferating rate in the GIST T1 cell line, upregulated pfetin expression at both the mRNA and protein level, suggesting that *KCTD12* expression is negatively influenced by KIT. These findings led us to hypothesize that high expression of pfetin in GIST clinical samples might be indicative of lower expression levels of KIT. In translocation sarcomas, such as Ewing’s sarcoma and synovial sarcoma, in which the chimeric fusion protein genes are thought to be oncogenes that play a central role in tumorigenesis, it has been shown that knockdown of those fusion genes decreased cell proliferation and induced apoptosis [32–34]. In GISTs, KIT may have the same function as the chimeric fusion proteins in these tumors. Pfetin expression in GIST may be indicative of lower *KIT* expression levels, and thus, could be considered a useful prognostic marker. However, because gene expression changes associated with KIT knock down would be stronger, a possibility remains that pfetin upregulation would simply be reflecting this global effect.

Finally, knockdown of *KCTD12*, which increased the cell proliferation rate in GIST T1 cells, reduced KIT expression at both the mRNA and protein level. These paradoxical relationships are currently difficult to explain. However, it is possible that these two proteins might collectively control the malignant potential of GISTs by regulating the cell proliferating rate in such a way that excessive GIST growth is limited, and GIST cells are prevented from excessive cell death and apoptosis.

In summary, pfetin functions as a tumor suppressor in GISTs, potentially by affecting the rate of cell proliferation. Pfetin expression in GIST is regulated by KIT and higher expression of pfetin was found to be indicative of lower *KIT* expression levels. Because KIT has a central role in GIST tumorigenesis, the expression level of pfetin is a promising prognostic marker.

## MATERIALS AND METHODS

### Patients

In total, 76 patient reports of primary GISTs were obtained from the files of the Department of Human Pathology, Juntendo University Hospital, Tokyo, Japan. These consecutive patients had been treated at the Juntendo University Hospital in the period between 2000 and 2010. With one exception, all patients were successfully treated surgically and were not given adjuvant treatments, such as imatinib mesylate before surgery. In the case 26, only a partial resection was performed due to large tumor size. Diagnosis was based on the WHO classification system for soft-tissue tumors [35]. In addition, diagnosis of GIST was confirmed by the immunohistochemical analysis with antibodies against the following proteins: c-kit (CD117 antibody, DAKO Japan Corp., Tokyo, Japan), CD34 (QBEnd/10, Leica Biosystems, New Castle, UK), DOG1 (K9, Leica Biosystems, New Castle, UK), and SDHB (21A11AE7, Abcam, Cambridge, UK). We used the following parameters for the risk classification: tumor site, tumor size, presence of necrosis, and mitotic rate [36]. Clinicopathological data of the 76 patient cases of GISTs are summarized in Supplementary Table 6. The institutional review board of Juntendo University hospital approved this study (permission No. 2012118). All experiments were performed in accordance with relevant guidelines and regulations. The methods were carried out in “accordance” with the approved guidelines. Written informed consent was obtained from all subjects.

### Mutational analysis of the *KCTD12* gene

Genomic DNA was extracted from each formalin-fixed paraffin-embedded (FFPE) tumor tissue-containing block. The primary tumor samples were selected for the aforementioned immunohistochemical and mutation analysis, where it was possible. Mutational analysis was performed for the entire region of the open reading frame of *KCTD12* in all 76 cases. We used the GeneRead DNA FFPE Kit (Qiagen) for DNA extraction to minimize the artificial effects derived from FFPE samples. In addition, when the mutations were detected, we confirmed the reproducibility of the results by second PCR amplification and sequencing. Furthermore, for the cases in which *KCTD12* mutations were detected, genomic DNA was also extracted from the corresponding non-tumor tissue surrounding the tumor in order to investigate whether these mutations were somatic. Primer sequences used in this study are described in Supplementary Table 5.

### Immunohistochemical analysis

Pfetin and KIT expression was examined immunohistochemically using paraffin-embedded tissues as described previously [6]. Briefly, tissue sections (4-mm thick) were autoclaved in a 10 mM citrate buffer (pH 6.0) at 121° C for 30 min and then incubated with our self-designed No. 10-4 anti-pfetin antibody (1:1000 dilution) [7] and anti-c-kit antibody (CD117 antibody, DAKO Japan Corp., Tokyo, Japan. 1:200 dilution). Immunostaining was performed according to the universal immunoperoxidase polymer method using Envision^+^ system-HRP (DAKO, Glostrup, Denmark). Two of the authors (T.O. and T.S.) examined the stained tissues and were blind to the clinical data. Any discrepancies were resolved by re-evaluation to reach a consensus. Regarding pfetin expression, as in our previous report [6], tumor cells were classified as stained if the pfetin staining intensity was higher than that of the vascular endothelial cells that served as an internal positive control in the same tissue section. Samples in which >20% of tumor cells were stained were considered to be pfetin-positive. Regarding the evaluation of c-kit expression level, focal/weak expression was scored as +; diffuse/weak expression as 2+; diffuse/strong expression as 3+.

### Knockdown of *KCTD12* and KIT in the GIST T1 cell line

To evaluate the function of endogenous pfetin and its possible cooperative action with KIT in GIST, we performed RNA interference experiments using siRNA duplexes against *KCTD12* and *KIT*. The GIST T1 cell line was kindly provided by Dr. T. Taguchi [37]. This cell line has a 57-bp deletion in exon 11 of *KIT* [37] and we confirmed the authenticity of this T1 cell line with 57-bp deletion. Briefly, 24 h before transfection, cells at 80% confluence were trypsinized and diluted with fresh medium without antibiotics to a concentration of 3 × 10^5^ cells/mL and then were transferred into either 6-well plates (2.5 mL/well) or a 96-well plate (0.1 mL/well). Transfection with 2 different siRNAs for each target: *KIT* (SASI_Hs01_00088058, SASI_Hs01_00088060, Sigma-Aldrich, MO, USA), KCTD12 (SASI_Hs01_00206464, SASI_Hs01_00206465, Sigma-Aldrich), and a scrambled siRNA as a negative control (Sigma-Aldrich) was carried out using Lipofectamine™ RNAiMAX reagent (Invitrogen, CA, USA) and 30 pmol of each siRNA duplex. Cells were harvested at 24, 48, 72, and 96 h after transfection and then analyzed by western blotting and the proliferation assay.

### RNA extraction and real-time PCR

Total RNA was extracted from cell pellets using TRIzol Reagent (Gibco/BRL, Tokyo, Japan) according to the manufacturer’s protocol. Five micrograms of RNA of each sample were used for the subsequent reverse transcription reaction (SuperScriptII) (Thermo Fisher Scientific, CA, USA). A semi-quantitative PCR was performed for *KIT and KCTD12* using a StepOne Real-Time PCR System (Applied Biosystems, CA, USA) and the predeveloped TaqMan assay reagents for *KIT* (Hs00174029_m1, Applied Biosystems CA, USA) and *KCTD12* (Hs00540818_s1, Applied Biosystems CA, USA). Human *TBP* was used as an endogenous control (Human *TBP* Endogenous Control, 4333769F, Applied Biosystems CA, USA). The comparative C_T_ (ΔΔC_T_) method was used for the semi-quantification of the PCR samples. The mRNA expression levels were normalized to that of the control sample after normalization by TBP at each time point.

### Mutation analysis of the KIT

Genomic DNA was extracted from each of the XX formalin-fixed and paraffin-embedded GIST samples. Mutation analysis of KIT was performed from exons 9, 11, 13, and 17 by PCR and direct sequencing. PCR cycle conditions were as follows: 94° C for 2 min followed by 40 cycles of 94° C for 30 s, 55° C for 30 s, and 72° C for 30 s, and a final hold at 72° C for 2 min. The primer sequences used are listed in Supplementary Table 5.

### Cell proliferation assay

Cell counting at each time point was performed in triplicate using Cell Counting Kit-8 (Dojindo, Tokyo, Japan) according to the manufacturer’s protocol.

### Invasion assay

The invasion assays were performed using 24-well BD BioCoat Matrigel Invasion Chambers (BD Biosciences, NY, USA) according to the manufacturer’s protocol. T1 cell suspensions were prepared at a density of 3 × 10^5^ cells/mL in 0.5 mL of the serum-free medium and added to the gel chamber insert. In 48 h after transfection with *KCTD12* siRNA, non-invading cells were removed with cotton swabs, invading cells were stained using Diff-Quick reagent (Sysmex, Hyogo Prefecture, Japan), and then the number of invading cells was counted.

### Microarray analysis

cDNA microarray analysis (Affymetrix GeneChip Human Genome U133 Plus 2.0 Array) was also performed to examine the global gene expression changes caused by the knockdown of *KIT* and *KCTD12* by using total RNA extracted 72 h after siRNA transfection. Lists of genes commonly up- or downregulated by the two different siRNAs were made for *KIT* and *KCTD12*, respectively. The gene ontology (GO) analysis was performed to evaluate the gene expression changes by each functional category.

### Western blot

Western blotting was performed after preparation of cell lysates in the radioimmunoprecipitation assay buffer. Nitrocellulose membranes were pre-incubated with 5% non-fat dry milk in Tris-buffered saline and Tween 20 (TBS-T) before their incubation with specific primary antibodies for 2 h. Bound molecules were visualized with horseradish peroxidase-conjugated anti-mouse or anti-rabbit secondary antibodies and enhanced chemiluminescence (Amersham Biosciences, Buckinghamshire, UK). We used primary antibodies against the following proteins: c-kit (sc-168, 1:500, Santa Cruz Biotechnology, Inc., TX, USA), KCTD12 (sc-84335, 1:500, Santa Cruz Biotechnology, Inc.), GAPDH (sc-32233, 1:1000, Santa Cruz Biotechnology, Inc.).

### Statistical analysis

The chi-square test (χ^2^) was used to establish an association between the presence of any *KCTD12* genetic mutation and corresponding protein expression. The impact of the *KCTD12* mutation and pfetin expression on the disease-free or overall survival was calculated using the Kaplan–Meier analysis with the log-rank test. The Mann–Whitney *U*-test was used to assess the relationship between the expression levels of *KCTD12* and *KIT*.

## SUPPLEMENTARY MATERIALS FIGURES AND TABLES








