# Supplementary material for: KCTD12 is negatively regulated by Kit in gastrointestinal stromal tumors
Source: Oncotarget. 2018 Jun 5;9(43):27016–26. doi: 10.18632/oncotarget.25469 (PMC6007475; doi:10.18632/oncotarget.25469)
Supplement: Supplementary file 1 [file oncotarget-09-27016-s001.pdf]

KCTD12 is negatively regulated by Kit in gastrointestinal stromal tumors

SUPPLEMENTARY MATERIALS

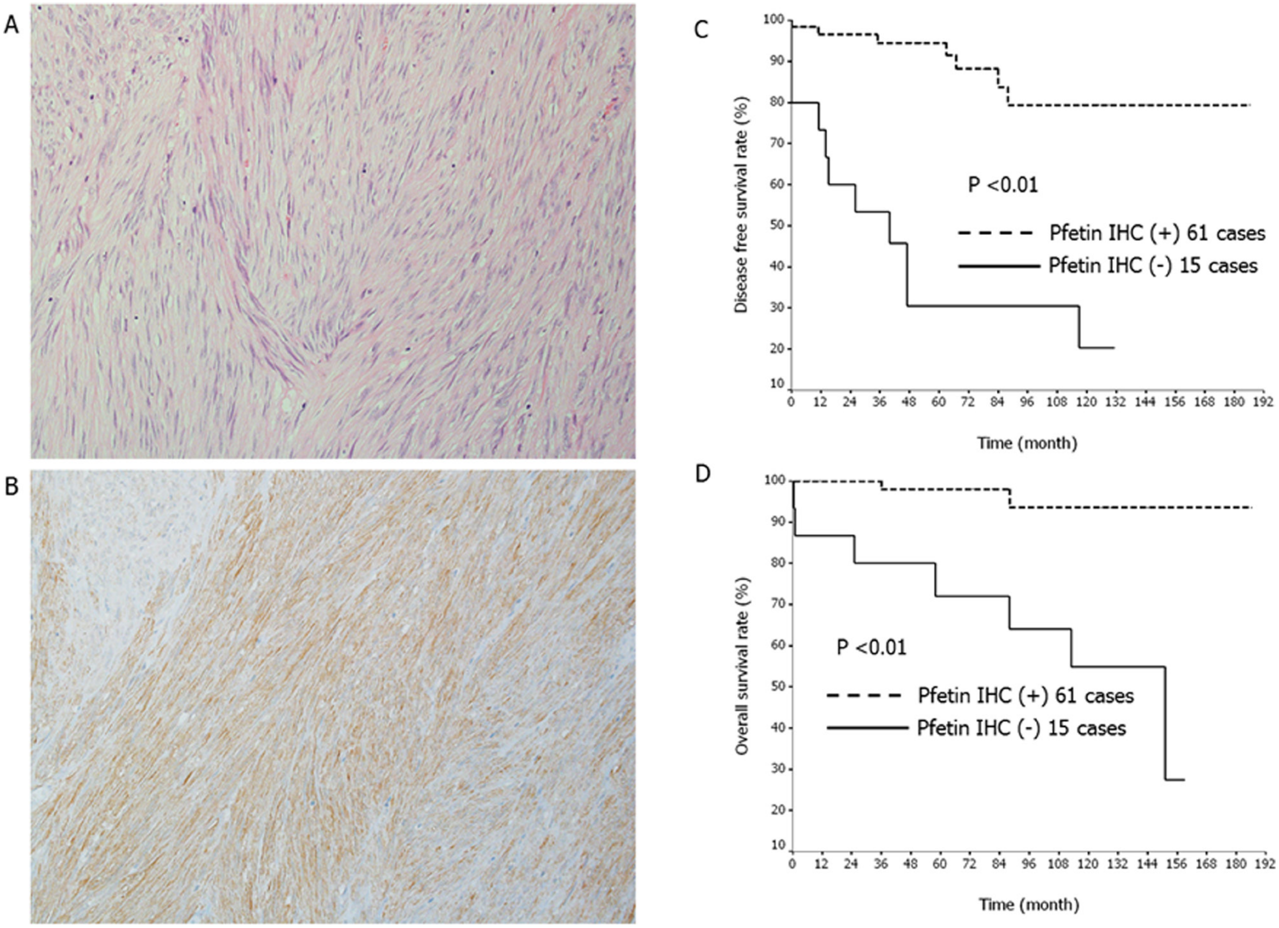

Supplementary Figure 1: Survival analysis according to the status of pfetin positivity or *KCTD12* genetic alteration. (**A**) Histology of a case of gastric GIST. (**B**) Immunohistochemistry for pfetin. Pfetin is diffusely positive for tumor cells. (**C**) Disease-free survival rate was significantly higher in pfetin-positive cases than in pfetin-negative cases. (**D**) Pfetin positivity significantly affected the overall survival in GIST patients.

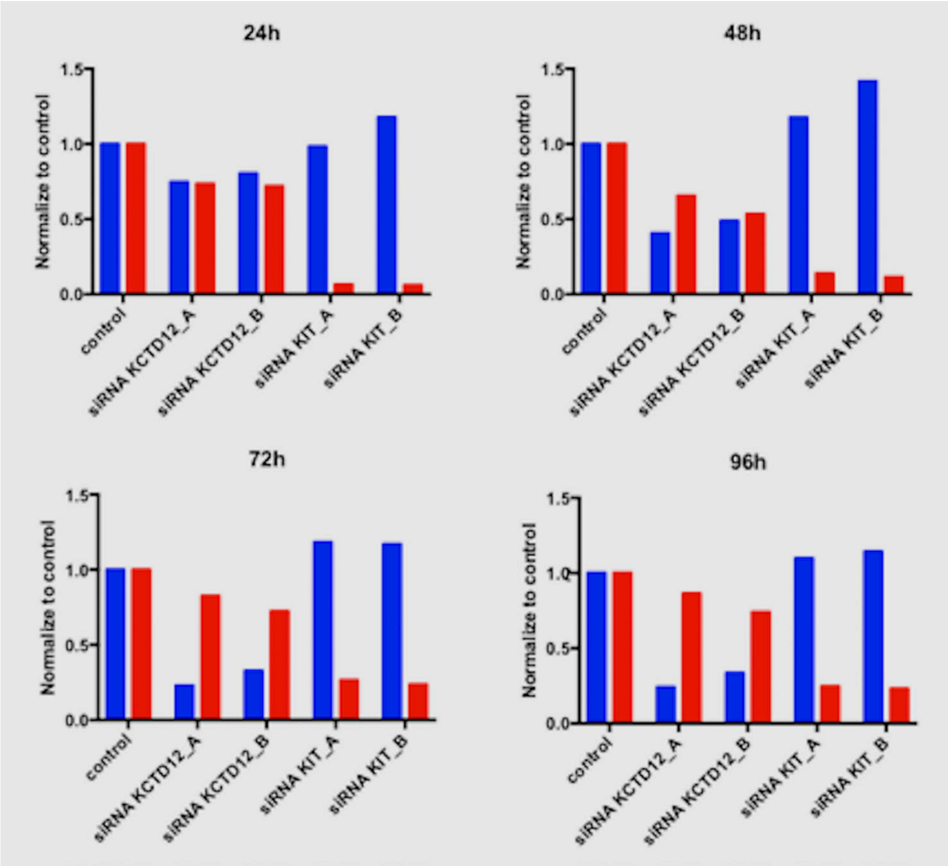

Supplementary Figure 2: Signal intensities of western blotting in Figure 3 were normalized by those of internal control GAPDH. Signal intensities were measured by soft-wares Image J. Blue: pfetin expression level. Red: Kit expression level.

Supplementary Table 1: Clinicopathologic characteristics of 76 GISTs

Features		No. of cases	Total
Age			76
	<50	20	
	50<	56	
Gender			76
	Female	28	
	male	48	
Site			76
	Stomach/Duodenum	60	
	Small intestine	10	
	Other	6	
Size			76
	<5 cm	44	
	5 cm–10 cm	24	
	10 cm<	8	
Necrosis			76
	–	54	
	+	22	
Mitosis			76
	<5	51	
	5–10	13	
	10<	12	
Risk ckassification			76
	Very low or low	38	
	Intermediate	10	
	High	28	
Pfetin expression			76
	Negative	15	
	Positive	61	
Pfetin mutation			76
	Mutaion	22	
	Wild type	54	
KIT expression			76
	Negative/Not informative	4	
	Positive	72	
KIT genotype			76
	Exon 9	2	
	Exon 11	56	
	Exon 13	2	
	Exon 17	3	
	N.A.	13	

N.A.: Not available for information

Supplementary Table 2: Association between pfetin expressions and co-factors

		Pfetin expression		Total	*P*-value
		Positive	Negative		
Total		61	15	76	
KCTD12 mutation					
	Positive	18	4	22	0.923
	Wild type	43	11	54	
KIT expression					
(***n*** = 72)	+	35	8	71	1.000
	2+/3+	24	5	1	
KIT mutation					
(***n*** = 69)	Exon 9	2	0	2	0.706
	Exon 11	46	10	56	
	Exon 13	1	1	2	
	Exon 17	3	0	3	
	N.A.	2	4	13	
Mitosis index					
(/50HPFs)	<5	45	6	51	0.029
	>5	16	9	27	
Risk classification					
	Very low or low	33	5	38	0.651
	Intermediate	8	2	10	
	High	20	8	28	

Statistical analysis by Chi-squared test.

N.A. data not avialable.

Supplementary Table 3: Association between *KCTD12* mutations and co-factors

		KCTD12 Mutation		Total	*P* value
		Mut	Wild		
		22	54	76	
KIT expression					
(***n*** = 72)	+	12	31	43	0.608
	2+/3+	10	19	29	
KIT mutation					
(***n*** = 76)	Exon 9	1	1	2	0.918
	Exon 11	16	40	56	
	Exon 13	0	2	2	
	Exon 17	1	2	3	
	N.A.	4	9	13	
Mitosis index					
	<5	14	37	51	0.789
	>5	8	17	25	
Size					
	<5 cm	13	31	44	0.685
	5 cm–10 cm	8	16	24	
	10 cm<	1	7	8	
Necrosis					
	−	17	37	54	0.666
	+	5	17	22	
Risk classification					
	Very low or low	12	26	38	0.084
	Intermediate	0	10	10	
	High	10	18	28	
Site					
	Stomach/ Duodenum	18	42	60	0.870
	Small intestine	3	7	10	
	Other	1	5	6	

Statistical analysis by Chi-squared test.

N.A.: Data not available.

Supplementary Table 4-1: The gene ontology (GO) analysis was performed to evaluate the gene expression changes by each functional category. Gene set names affected by the knockdown of KCTD12 or KIT in GIST T1 cell are shown in these Tables, respectively. Activated gene sets by knockdown are highlighted in red, and suppressed gene sets are highlighted in blue. See Supplementary_Table_4-1

Supplementary Table 4-2: The gene ontology (GO) analysis was performed to evaluate the gene expression changes by each functional category. Gene set names affected by the knockdown of KCTD12 or KIT in GIST T1 cell are shown in these Tables, respectively. Activated gene sets by knockdown are highlighted in red, and suppressed gene sets are highlighted in blue. See Supplementary_Table_4-2

Supplementary Table 5: Primer seqences for *KCTD12* and KIT

KCTD12	
1F	GAAGGCGGTTGCAGCTCCTGAG
1R	AGCGCCGGGTCACGTACACCT
2F	GACAGCAAAGGCCGCTTCTT
2R	CACGAGCTCTGGCAGCTCGAA
3F	CGGGACTTGCAGCTCGTGCTG
3R	TCACCCAGCGAGCCCTCCTTGTG
4F	AGTACTTCGAGCTGCCAGAG
4R	CTGTTCGGGCTCCGAGTAGC
5F	GTGACGAGCTGCTGCCGCTT
5R	CCGATGGTGATGTAGCCCGAG
6F	GGGCTACATCACCATCGGCT
6R	GGCTTTCGTTCAGGGTGTCC
7F	ATCACCGTTTGCGGAAAGACG
7R	CACCATGTGGAAGCCCGACTC
8F	GCCTTCGACAAGCTGTCCGA
8R	GGAGCTCACTCCCTGCAGAA
KIT	
Ex9-F	GCCACATCCCAAGTGTTTTATG
EX9-R	GAGCCTAAACATCCCCTTAAATTG
Ex11-F	CCAGAGTGCTCTAATGACTG
EX11-R	AGCCCCTGTTTCATACTGAC
Ex13-F	CTTGACATCAGTTTGCCAGTTGT
EX13-R	GACAGACAATAAAAGGCAGCTTG
Ex17-F	TGGTTTTCTTTTCTCCTCCAA
EX17-R	GCAGGACTGTCAAGCAGAGA

Supplementary Table 6: Clinicopathologic features of the 76 GIST cases. See Supplementary_Table_6
